# Integra^®^ Dermal Regeneration Template in Complex Scalp Reconstruction

**DOI:** 10.3390/jcm13051511

**Published:** 2024-03-06

**Authors:** Natalie Turton, Aaina Aggarwal, Eoin Twohig, James Gallagher, Kieron McVeigh, Neal Barnard, Karl Payne

**Affiliations:** 1 Worcestershire Acute Hospitals NHS Trust, Worcestershire WR5 1DD, UK; aaina.aggarwal@nhs.net (A.A.); eoin.twohig@nhs.net (E.T.); jamesgallagher@nhs.net (J.G.); kieron.mcveigh1@nhs.net (K.M.); neal.barnard@nhs.net (N.B.); k.payne.1@bham.ac.uk (K.P.); 2 Birmingham Medical School, University of Birmingham, Birmingham B15 2TT, UK; 3 Institute of Cancer and Genomic Sciences, University of Birmingham, Birmingham B15 2TT, UK

**Keywords:** Integra^®^, scalp defects, skin graft, scalp reconstruction, skin cancer, complications

## Abstract

**Background/Objectives**: The need for surgical reconstruction of scalp defects following the excision of cutaneous skin cancers is an increasingly common procedure. Particular challenges arise when considering options for reconstruction of large defects not amenable to local skin flap coverage. The use of skin grafts poses the risk of donor site morbidity. This paper investigates the emerging use of Integra^®^, a synthetic acellular dermal regeneration template, as an alternative or adjunct to skin grafting in scalp reconstruction. **Methods**: The study presents a retrospective analysis of 101 patients who underwent Integra^®^-based reconstruction of scalp defects. Demographics, procedure details, complications, need for further surgery, and time to healing were evaluated. **Results**: The overall success rate of the one-stage Integra^®^-only procedure was 95%, with a minor complication rate of 30.7%. Anticoagulation medication was identified as an independent risk factor for post-operative infection, while previous head and neck radiotherapy and increased defect depth were associated with the requirement for a second-stage skin graft. **Conclusions**: These findings support the consideration of Integra^®^ as a safe and viable alternative for both partial and full thickness scalp defects in a select cohort of complex highly co-morbid patients, reducing complications and the need for additional procedures.

## 1. Introduction

Surgical reconstruction of large scalp defects, most commonly as a result of the excision of cutaneous skin cancers, is a continued challenge to reconstructive surgeons. When evaluating reconstructive options, defects are typically classified as partial thickness or full thickness. Partial thickness defects, which involve the preservation of the pericranium and not amenable to local flap closure, can often be reconstructed using split or full thickness skin grafts. However, full thickness defects pose a greater challenge, and conventional reconstructive options include extensive local flaps, free-tissue transfer or skin grafts with bone burring to stimulate angiogenesis—all of which may not always be feasible in complex cases [1,2]. Donor site morbidity from skin grafts and the difficulties associated with local skin flap closure for specific defects must be carefully considered. Where feasible, it is prudent to opt for the most straightforward approach to achieve a scalp reconstruction that is both functional and aesthetically pleasing, whilst minimising complexity [2]. A particular set of patients pose the greatest challenge in scalp reconstruction, specifically the following: elderly and highly co-morbid patients, those with poor local skin quality, field change unamenable to local skin flaps, full thickness defects with exposed bone, and a potential lack of donor sites for tissue transfer in addition to those who are not fit for general anaesthetic or prolonged procedures.

Originally developed for burn wound coverage, Integra^®^ is a synthetic acellular dermal regeneration template that promotes revascularisation and connective tissue formation [3]. The emerging use of Integra^®^ as an adjunct or alternative to skin grafting offers potential solutions to the many challenges in scalp reconstruction, as well as a unique approach to the reconstruction of full thickness defects [4]. Multiple studies have demonstrated the effectiveness of Integra^®^ in scalp reconstruction [4,5,6], as well as its expanding applications in various anatomical locations. The current literature indicates favourable outcomes and low complication rates associated with the use of Integra^®^ across different surgical specialties [4,5,6,7,8,9,10,11].

The majority of evidence in the current literature focuses on analysing the outcomes of Integra^®^ in scalp reconstruction primarily as a two-stage procedure, involving a combination of full and partial thickness defects. This procedure involves the placement of Integra^®^ immediately following tumour resection, followed shortly thereafter by the placement of a split-thickness skin graft. A study by Tufaro et al. in 2007 demonstrated favourable outcomes with this two-stage technique across various anatomical locations, including in the head and neck, with 94% of patients achieving 100% skin graft uptake [7].

According to the published recommendations from a multidisciplinary advisory board in Italy in 2019, Integra^®^ is recommended for elderly patients with multiple comorbidities who have a higher risk for potential complications in traditional surgery [8]. Various studies have demonstrated favourable outcomes with the use of Integra^®^ as a one-stage procedure in the head and neck, both in scalp reconstruction as well as in deep facial defects [9,10,11]. These patients can benefit from reduced anaesthetic risk, reduced operating time, simplified post-surgical care and limited morbidity at the donor site with acceptable functional and aesthetic post-operative results [11].

This study presents data from a large case series using Integra^®^ as a single-stage reconstructive option in both partial and full thickness defects. Our aim is to investigate if Integra^®^ alone can be used to reconstruct large complex scalp defects, both partial and full thickness, thus mitigating the anaesthetic and donor site risk of a second-stage skin graft.

## 2. Methods

Outcomes from 101 patients over a 5-year period (January 2018 to December 2022) were retrospectively reviewed. All 101 patients underwent a standard surgical procedure, which involved either primary lesion excision, followed by immediate placement of Integra^®^, or solely as a secondary reconstruction procedure whereby Integra^®^ was being used to reconstruct a pre-existing defect. The Integra^®^ Dermal Regeneration Template, a two-layer skin regeneration system, was the material of choice used in this study [3]. The procedure was carried out by one of six consultant surgeons within a single department. Following an appropriate oncologically sound surgical excision, a 5–10 mm cuff of peripheral periosteum was left to aid healing. The Integra^®^ was placed onto the defect and secured on the periphery with sutures. A non-adherent dressing and double layer foam dressing was secured over the Integra^®^. Patients were followed up at 1 week post-operatively to review the dressing with dressing removal at 3–4 weeks post-operatively. In the event of suspected infection, broad-spectrum antibiotics according to hospital formulary guidelines were used and then tailored specifically to the patient once microscopy culture and sensitivity results became available.

Data were collected on various variables, including patient demographics, procedure details, histopathology, and postoperative outcomes, such as complications, management of complications, the need for further surgery, and time to final healing. Data were collected from patient records and recorded anonymously. Statistical analysis was performed using GraphPad PRISM (version 10.2.0), utilising linear regression, multiple logistic regression modelling, and chi-squared analysis. A normal distribution of data was assumed, using a line of best fit for linear regression. Post-operative complication (yes or no) as a binary outcome was used as the dependent variable for multiple logistic regression, using a Poisson regression model for categorical variables. Two-by-two contingency tables were used to perform a chi-squared analysis at a significance level of *p* < 0.05 for various combinations of variables and outcomes.

## 3. Results

### 3.1. Patient Demographics

An overview of patient clinical demographics can be found in Table 1. The majority of patients were male with an average age of 80.5 years (range 52–99 years). Most patients had a Charlson Comorbidity Index (CCI) score greater than or equal to 5, this represented 69% of the cohort, indicating severe or very severe comorbid disease with a predicted 10-year survival of <20%. This is consistent with the expected demographic for this procedure in an elderly population with significant comorbidities. Common comorbidities included diabetes, active or previous cancer, anti-thrombotic medication use and concurrent immunosuppression.

### 3.2. Procedure Details

**Figure 1:**
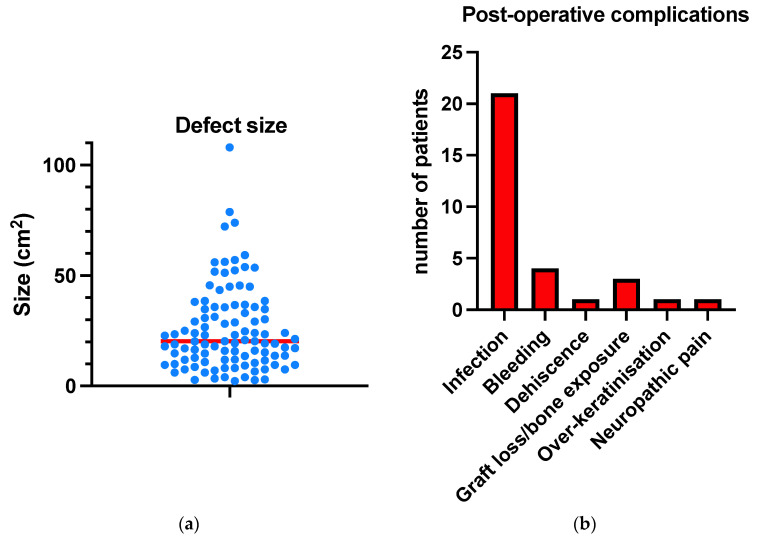
Graphs demonstrating (**a**) the distribution of defect sizes (cm^2^) observed in the cohort, with the average defect size highlighted in red and (**b**) the frequency of post-operative complications observed.

Table 2 details the procedure and pathology characteristics. The majority of defects resulted from the resection of malignant skin lesions, most commonly squamous cell carcinomas (58%) and basal cell carcinomas (6.9%). The average defect size was 25.5 cm^2^, with a range of sizes observed, including both small defects (0.1–20 cm^2^) and larger defects extending up to 100 cm^2^ (Figure 1a). In terms of depth, 37 defects were full thickness, involving the pericranium, while 64 were partial thickness, preserving the pericranium. As expected, greater than 80% of resections were for a malignant skin cancer diagnosis (*n* = 83).

### 3.3. Post-Operative Complications

Table 3 details surgical outcome and complication data. The incidence of post-operative complications was low, considering the elderly comorbid population. The most common complication was infection, observed in 21 of the 101 cases (20.8%), all of which were managed non-surgically using oral or topical antimicrobials. The second most common complication was minor bleeding, observed in four cases, followed by graft loss and bone exposure (Figure 1b).

### 3.4. Further Surgery Requirement

Among the 101 patients, only 5 required a second-stage procedure involving a skin graft, resulting in an overall success rate of 95% for the one-stage Integra^®^-only procedure. Among these five patients, four underwent reconstruction with a split-thickness skin graft, while one required a full thickness skin graft. The most common reason for the need for further surgery was persistent bone exposure and non-healing. For cases requiring a second stage, the average time to further surgery was 3.2 months.

### 3.5. Overall Outcome

**Figure 2:**
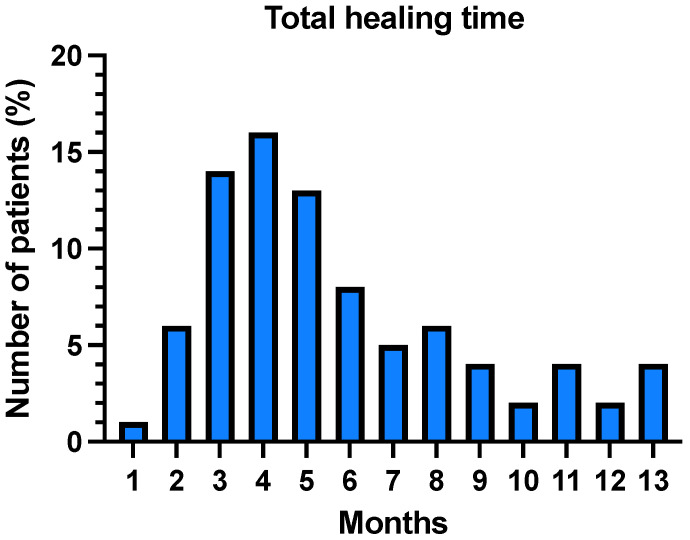
Bar chart demonstrating the distribution of healing times in months.

The median time to full recovery (defined as full defect coverage with complete healing) was 4 months. However, the distribution of healing times indicated that a large proportion of patients achieved full healing within 4 months (Figure 2) with a select few highly co-morbid patients requiring up to 1 year of dressings. Surgical complications were significantly associated with prolonged recovery times, as determined by multiple linear regression modelling (*p* = 0.0022). Categorising the final outcomes, 85 defects were classified as acceptable healing and five cases required a second-stage skin graft. Sixteen cases were inconclusive due to incomplete follow-up data, such as cases involving patient death or early removal of Integra^®^ due to involved margins or recurrence at the site.

**Figure 3:**
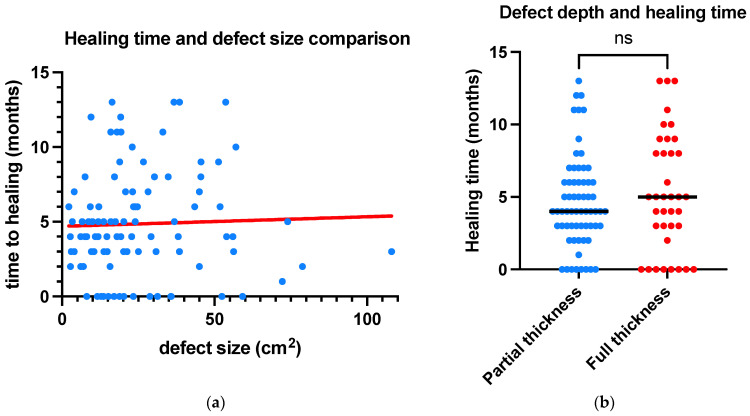
Graphs demonstrating outcomes of statistical analysis for (**a**) simple linear regression modelling of time to healing and defect size and (**b**) *t*-test for time to healing and defect thickness (ns = not-significant).

**Figure 4:**
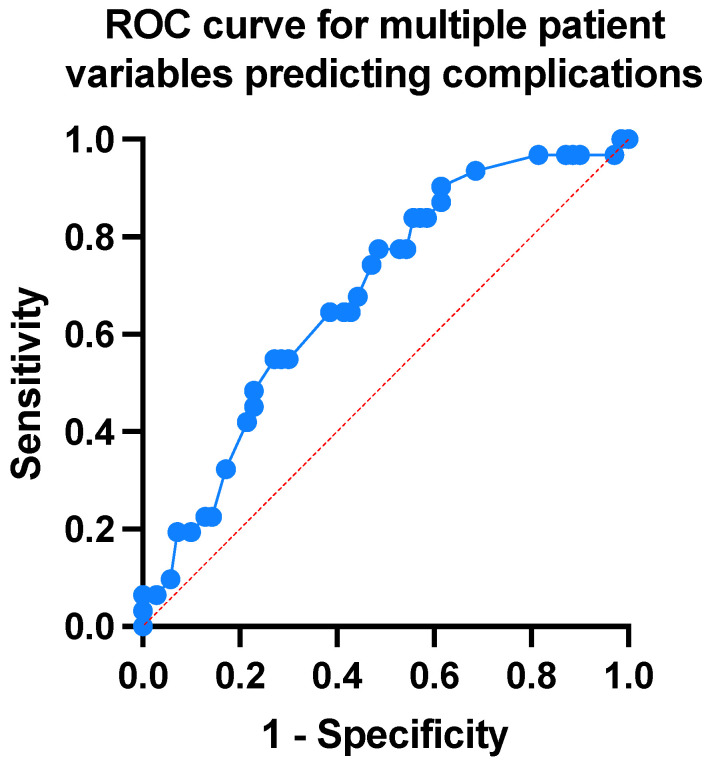
Multivariate modelling to predict complications. All recorded patient variables were combined into a multiple logistic regression model to predict complication rate following Integra^®^ reconstruction. AUC equals 0.68, with an NPV of 72.22% and PPV of 54.55% (*p* = 0.0039). The data demonstrates that patient variables are more beneficial inidentifying those who will not suffer complications, but is an overall poor predictor of complications, i.e., likely confounding factors associated with defect depth and size (AUC = area under curve, NPV = negative predictive value, PPV = positive predictive value).

Simple linear regression analysis revealed no correlation between defect size and time to healing (*p* = 0.732), a finding replicated when subgroup analysis of partial and full thickness defects was conducted (Figure 3). Patient factors (co-morbidity index, previous radiotherapy, immunosuppression, smoking, and anticoagulation) were modelled using multiple logistic regression to determine the utility of pre-operative variables to predict a post-operative complication. Together, they demonstrated an AUC of 0.68, with an NPV of 72%, and PPV of 55%. However, among these variables, patients taking anticoagulation was identified as an independent risk factor for a complication, which on closer examination was found to be linked to post-operative infection with an odds ratio of 3.2 (95% CI 1.126–9.975). No other demographic, operative, or pathological factors were found to increase the overall complication rate (Figure 4).

Further statistical analysis was used to examine different combinations of variables and outcomes. This analysis revealed associations between previous head and neck radiotherapy (*p* = <0.00001, chi-squared) and depth of the defect (*p* = 0.039, chi-squared) with the requirement for a second-stage skin graft, which were significant at *p* < 0.05. Among the five patients requiring a second-stage skin graft, four had full thickness defects, and three had undergone previous head and neck radiotherapy. Further analysis focused on the partial- and full-thickness-defect cohorts, which yielded mean healing times of 4.6 months and 5.3 months, respectively, and this was not found to be statistically significant (*p* = 0.474, chi-squared). No variables were found to be significantly associated with the development of post-operative complications or impaired healing. Furthermore, defect size was not found to impact the risk of developing post-operative complications (*p* = 0.912, chi-squared), requirement for second-stage surgery (*p* = 0.946, chi-squared) or impaired healing (*p* = 0.34, chi-squared).

**Figure 5:**
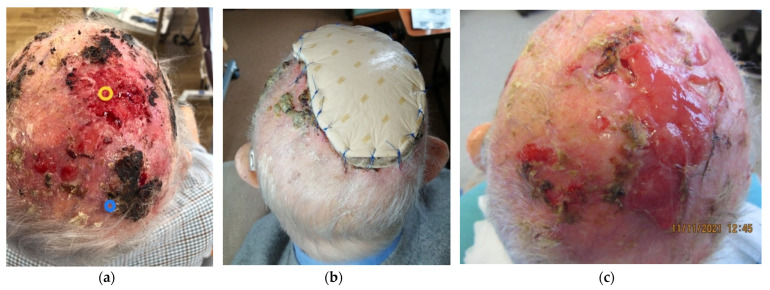
Clinical photographs of a 93-year-old male patient treated in 2021 who underwent excision of an extensive squamous cell carcinoma (SCC) of the scalp, with the resultant full thickness defect being reconstructed with Integra^®^ as a one-stage procedure. (**a**) Pre-operative image of the scalp SCC. (**b**) Integra^®^ with overlying foam dressing 1 week post-operatively. (**c**) Wound granulation at 7 months post-operatively.

**Figure 6:**
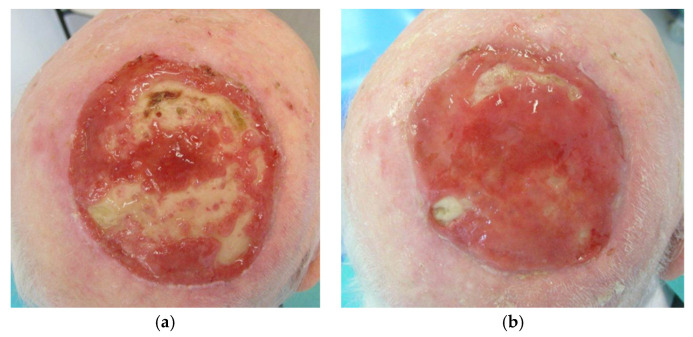
Clinical photographs of a large full thickness scalp defect reconstructed with Integra^®^ as a single-stage procedure at (**a**) 6 weeks post-operatively, with partial granulation over the bone and again at (**b**) 10 weeks post-operatively, with the majority of bone now covered with ongoing granulation.

**Figure 7:**
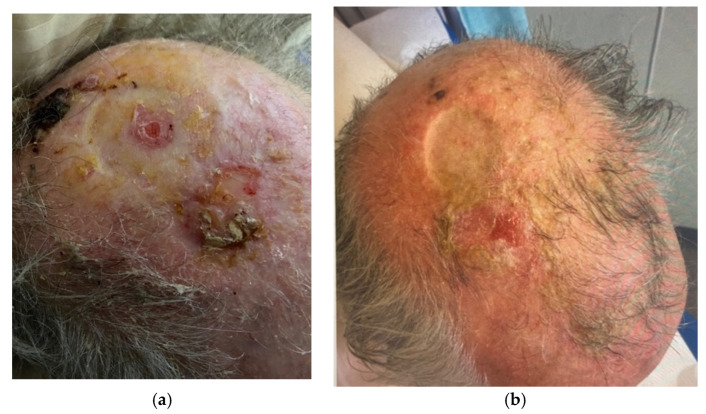
Clinical photographs demonstrating complete healing of a smaller full thickness defect reconstructed with Integra^®^ as a one-stage procedure at (**a**) 4 months and (**b**) 5 months post-operatively.

**Figure 8:**
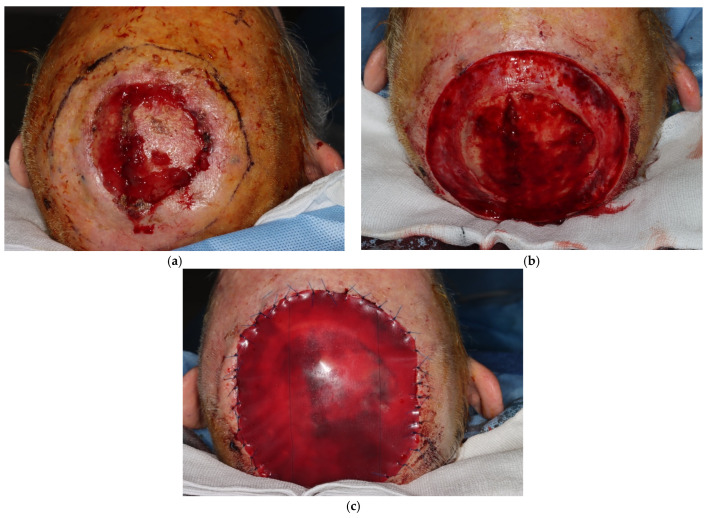
Clinical photographs demonstrating single-stage Integra^®^ use in a large scalp defect, (**a**) pre-operatively showing extent of the lesion (**b**) post-excision and (**c**) with Integra^®^ bilayer regeneration template sutured into place, covering the final defect. The defect healed successfully but unfortunately the patient suffered recurrence.

Clinical photographs (Figure 5, Figure 6, Figure 7 and Figure 8) demonstrate the stages of wound healing defects reconstructed with Integra^®^ alone. These photographs show the progression of healing at different postoperative time points, highlighting the successful outcomes achieved.

## 4. Discussion

Scalp reconstruction poses significant challenges, particularly in complex co-morbid patients with advanced-stage cancers and large-sized defects. Integra^®^ is a dermal regeneration template with emerging use in both clinical practice and the scientific literature for its potential applications in various surgical areas [4]. This retrospective study examines the outcomes and limitations of scalp reconstruction using Integra^®^ in a cohort of 101 patients. This is the largest cohort of patients who underwent Integra^®^ reconstruction for scalp defects primarily as a one-stage procedure in the current literature, thus highlighting the versatility of Integra^®^ usage.

Integra^®^ demonstrates success in both full and partial thickness defects, even when used without any underlying periosteum, as demonstrated by Singh et al., where it was successfully used to provide soft tissue coverage over a segment of exposed mandible [12]. Furthermore, Integra^®^ has various applications in other areas of reconstructive surgery, demonstrating success in cases of burns and limb salvage surgery [13]. Additionally, Integra^®^ can be utilised as a single-stage or a two-stage procedure in conjunction with a split or full thickness skin graft. The majority of existing research focuses on Integra^®^ when combined with a second-stage skin graft. However, success has been demonstrated in case series by both De Angelis et al. [9] and Koenen et al. [10] on the use of Integra alone to reconstruct both scalp and facial defects without a second-stage skin graft.

With regard the application of Integra^®^ to scalp reconstruction, Mogedas-Vegara et al. published a study of 70 patients who underwent scalp tumour excision followed by a two-stage reconstruction with Integra^®^ and a split-thickness skin graft [5]. The study population had an average age of 83.3 years with 92.9% of patients classified as medically comorbid. The mean defect size was 23 cm^2^, with 94% being full thickness defects. The overall success rate was 87.1%, with Integra^®^ failure in nine patients. Romano et al. published a case series of 20 patients who underwent the same two-stage procedure in 2021 [6]. The median age of their patients was 63 years with a mean defect size of 72 cm^2^. Five of the twenty patients experienced complications, including delayed healing and infection. However, all 20 cases demonstrated skin graft take ranging from 95–100%.

In our study, scalp reconstruction using Integra^®^ demonstrated high success rates and low complication rates. Our infection rate of 20.8% aligns with the rate reported in Mogedas-Vegara’s study (18.6%), and our overall complication rate of 30.7% is comparable to the rate reported in Romano et al.’s paper (25%). However, these positive outcomes come at the expense of extended healing times and the need for multiple attendances for dressing changes and review. The median time to full healing was 4 months, indicating that patients and healthcare professionals should consider these factors when planning for scalp reconstruction using Integra^®^. Using a single-stage technique, scalp reconstruction with Integra^®^ yielded a positive outcome in 95% of patients, with only five patients requiring a second-stage skin graft procedure either due to ongoing unacceptable bone exposure or impaired healing. Therefore, despite longer healing times, overall patient morbidity is reduced through decreasing the number of clinical procedures required as well as a reduced hospital stay. A 10-year retrospective review of scalp reconstruction by Steiner et al. [14] reported an average post-operative hospital stay of 6.4 days in patients requiring a split-thickness skin graft; however, in our unit, all Integra^®^ cases are performed as a day case procedure under local anaesthetic. Corradino et al. also performed primary scalp tumour excision and Integra^®^ placement under a local anaesthetic, with an average operating time of 30.4 min [15]. Although the average operating time was not recorded in our study, the overwhelming majority of procedures were also performed under a local anaesthetic, thus further demonstrating that by avoiding a general anaesthetic, operating times and unnecessary anaesthetic-related complications in an already elderly co-morbid patient group can be reduced.

Of note, Modega et al. [5] commented on seven patients who did not undergo a second-stage split-thickness skin graft due to rapid epithelialisation of the Integra^®^ graft before the planned second-stage procedure. The authors suggested that a second stage may not always be necessary in some cases, hypothesising that this may be related to smaller defect sizes, as observed in their cases. However, in our study, defect size did not impact complication rates, healing time, or end result. Our data point towards patient factors as having greater significance—a key finding from this study and of importance during surgical planning.

Significantly, there was no notable difference in outcomes between partial and full thickness defects, indicating that Integra^®^ performs well even when directly applied onto bone. However, an increased depth of defect was found to be a risk factor for requiring a second-stage skin graft. To validate the efficacy of Integra^®^ use in full thickness defects, the study compared outcomes between the two groups, with no differences in complication rate, healing time, or final outcome identified. As highlighted, in terms of determining the success of scalp reconstruction, patient factors were found to have a greater impact than surgical factors. Most notably, this included associations between previous head and neck radiotherapy with a requirement for second-stage defect, as well as with antithrombotic medication identified as an independent risk factor for developing a complication. Individual patient characteristics appear to play a crucial role in determining the outcome of the procedure, highlighting the importance of personalised treatment approaches. Such an aspect is even more important when considering that this surgical approach may be utilised in the neoadjuvant setting. The emerging use of immunotherapy in select skin cancer patient cohorts, similar to those examined in our study, is an element to consider when planning treatment and assessing the impact of medical comorbidities on surgical outcomes and future treatment, as has been discussed elsewhere [16]—a potential avenue of future research.

As a retrospective study, it is accepted that this research is subject to inherent limitations. Specifically, the absence of a control group receiving traditional skin grafts does not allow for a direct comparison of outcomes between Integra^®^ and other techniques. Future studies should aim to explore both the effectiveness and healing times of Integra^®^ compared to conventional scalp reconstruction methods, such as traditional skin grafts, to provide a comprehensive understanding of its benefits and limitations in scalp reconstruction. Moreover, the healing times reported in the study are derived from the moment when patients were assessed in clinic and considered to be fully healed based on clinical observation. However, it is important to consider that due to the considerable intervals between appointments, these figures may have been overestimated as defects are likely to have already healed completely some time before the appointment date.

## 5. Conclusions

In summary, this study provides evidence from a large case series that Integra^®^ can be successfully used in scalp reconstruction in a single-stage procedure for both partial and full thickness defects. The study presents success rates greater than 90% with a minor complication of 30%. The key finding is that irrespective of defect size, patient factors have the greatest influence upon complication rate. These findings demonstrate the potential of Integra^®^ as an effective solution for scalp reconstruction in isolated cases, particularly among complex co-morbid patients with complex cancers and a wide range of defect sizes and stages.

Clearly, patient selection is of critical importance and the risk of increased healing time must be weighed against the decreased surgical morbidity of single-stage Integra^®^ reconstruction, with clear communication of this to the patient, along with informed consent. However, in highly co-morbid patients with extensive lesions or field change unamenable to local skin flaps [9], our evidence is promising for the use of Integra^®^ and significantly adds to the existing literature. Future research should seek to identify variables for patient selection or modifiable factors that could increase healing time and decrease complication rates in these Integra^®^-treated patients. Nevertheless, in a small subset of patients, Integra^®^ now represents a viable treatment option in previously difficult-to-treat defects. 

## Figures and Tables

**Table 1:** Table demonstrating summary of results for patient clinical demographics.

Variable	Characteristics	Frequency (*n* = 101)	Percentage (%)
Age (Years)	50–59	4	4
	60–69	8	8
	70–79	31	30.6
	80–89	43	42.5
	>90	15	14.9
Gender	Male	86	85.1
	Female	15	14.9
Charlson Comorbidity Index (CCI)	Mild (1–2)	6	5.9
	Moderate (3–4)	25	24.8
	Severe (5–6)	30	29.7
	Very Severe (7+)	40	39.6
Medical Comorbidities of Interest	Diabetes	22	21.8
	Active or Previous Cancer	45	44.6
	Previous H&N Radiotherapy	4	4
	Antithrombotic Drugs	48	47.5
	Immunosuppressants	22	21.8
Smoking History	Non-smoker	38	37.7
	Ex-smoker	56	55.4
	Current Smoker	7	6.9
Alcohol Use	None	33	32.7
	Light (1–7 units weekly)	48	47.5
	Moderate (8–14 units weekly)	11	10.9
	Heavy (>15 units weekly)	9	8.9

**Table 2:** Table demonstrating summary of results for procedure details and pathological details.

Variable	Characteristics	Frequency (*n* = 101)	Percentage (%)
Defect Site	Frontal	26	25.7
	Parietal	18	17.8
	Vertex	47	46.7
	Occipital	10	9.9
Defect Size (Diameter in cm^2^)	0.1–20 cm^2^	49	48.5
	20.1–40 cm^2^	34	33.7
	40.1–60 cm^2^	14	13.8
	60.1–80 cm^2^	3	3
	80.1–100 cm^2^	0	0
	100.1–120 cm^2^	1	1
Periosteum Resected in Specimen	Yes	37	36.6
	No	64	63.4
Pathological Diagnosis	Scar Tissue	7	6.9
	Actinic Keratosis	7	6.9
	SCC	59	58.5
	BCC	7	6.9
	Malignant Melanoma	6	5.9
	Other Malignant Diagnoses *	11	10.9
	Pre-existing Bony Defect	4	4
Tumour Stage	pT1	15	14.9
	pT2	19	18.8
	pT3	31	30.6
	pT4	1	1
	pT4N1	1	1
	No Staging Given	34	33.7
Tumour Grade	1	2	2
	2	36	35.6
	3	21	20.8
	No Grading Given	42	41.6

* Other malignant diagnoses: atypical fibroxanthoma (*n* = 3), pleomorphic dermal sarcoma (*n* = 6), angiosarcoma (*n* = 1) and malignant.

**Table 3:** Table demonstrating summary of results for patient outcomes, including post-operative complications, requirement for further surgery, total healing time, and end result.

Outcome	Characteristics	Frequency	Percentage
Post-Op Complication (*n* = 101)	Yes	31	30.6
	No	70	69.4
Complication Type (*n* = 31)	Localised Infection	21	67.7
	Bleeding	4	12.9
	Dehiscence	1	3.2
	Graft Loss/Bone Exposure	3	9.8
	Over-Keratinisation	1	3.2
	Neuropathic Pain	1	3.2
Second-Stage Surgery Required (*n* = 101)	Yes	5	5
	No	96	95
Second-Stage Surgery Type (*n* = 5)	STSG	4	80
	FTSG	1	20
Time Between Surgeries (*n* = 5)	<1 month	0	0
	>1–4 months	4	80
	>4–8 months	0	0
	>8–12 months	1	20
Total Healing Time (*n* = 101)	<1 month	1	1
	>1–4 months	36	35.6
	>4–8 months	32	31.7
	>8–12 months	12	11.9
	>12 months	4	4
	Inconclusive *	16	15.8
End Result (*n* = 101)	Full Healing	69	68.4
	Over-Granulation	7	6.9
	Exposed Bone	9	8.9
	Inconclusive	16	15.8

* Inconclusive due to incomplete follow-up data due to patient death, poor attendance or further surgery requiring early removal of Integra^®^ (due to involved margins or recurrence at site).

## Data Availability

The data presented in this study are available on request from the corresponding author.
